# Effects of sodium tanshinone IIA sulfonate injection on inflammatory factors and vascular endothelial function in patients with acute coronary syndrome undergoing percutaneous coronary intervention: A systematic review and meta-analysis of randomized clinical trials

**DOI:** 10.3389/fphar.2023.1144419

**Published:** 2023-03-07

**Authors:** Zunqi Kan, Wenli Yan, Mengqi Yang, Huanyu Gao, Dan Meng, Ning Wang, Yuqing Fang, Lingyu Wu, Yongmei Song

**Affiliations:** ^1^ College of Chinese Medicine, Shandong University of Traditional Chinese Medicine, Jinan, Shandong, China; ^2^ Institute for Literature and Culture of Chinese Medicine, Shandong University of Traditional Chinese Medicine, Jinan, Shandong, China

**Keywords:** sodium tanshinone IIA sulfonate, acute coronary syndrome, percutaneous coronary intervention, systematic review, meta-analysis

## Abstract

**Background:** Patients with acute coronary syndrome (ACS) undergoing percutaneous coronary intervention (PCI) therapy may experience further damage to the vascular endothelium, leading to increased inflammatory response and in-stent thrombosis. In many clinical studies, sodium tanshinone IIA sulfonate injection (STS) has been found to reduce inflammatory factors and enhance vascular endothelial function in patients with ACS while improving the prognosis of PCI. However, to date, there has been no systematic review assessing the effectiveness and safety of STS on inflammatory factors and vascular endothelial function.

**Purpose:** The aim of this study is to systematically review the effects of STS on inflammatory factors and endothelial function in patients with ACS treated with PCI.

**Methods:** Until October 2022, eight literature databases and two clinical trial registries were searched for randomized controlled trials (RCTs) investigating STS treatment for ACS patients undergoing PCI. The quality of the included studies was assessed using the Cochrane Risk Assessment Tool 2.0. Meta-analysis was performed using RevMan 5.4 software.

**Results:** Seventeen trials met the eligibility criteria, including 1,802 ACS patients undergoing PCI. The meta-analysis showed that STS significantly reduced high-sensitivity C-reactive protein (hs-CRP) levels (mean difference [MD = −2.35, 95% CI (−3.84, −0.86), *p* = 0.002], tumor necrosis factor-alpha (TNF-α) levels (standard mean difference [SMD = −3.29, 95%CI (−5.15, −1.42), *p* = 0,006], matrix metalloproteinase-9 (MMP-9) levels [MD = −16.24, 95%CI (−17.24, −15.24), *p* < 0.00001], and lipid peroxidation (LPO) levels [MD = −2.32, 95%CI (−2.70, −1.93), *p* < 0.00001], and increased superoxide dismutase (SOD) levels [SMD = 1.46, 95%CI (0.43, 2.49), *p* = 0,006] in patients with ACS. In addition, STS significantly decreased the incidence of major adverse cardiovascular events (relative risk = 0.54, 95%CI [0.44, 0.66], *p* < 0.00001). The quality of evidence for the outcomes was assessed to be very low to medium.

**Conclusion:** STS can safely and effectively reduce the levels of hs-CRP, TNF-α, MMP-9, and LPO and increase the level of SOD in patients with ACS treated with PCI. It can also reduce the incidence of adverse cardiovascular events. However, these findings require careful consideration due to the small number of included studies, high risk of bias, and low to moderate evidence. In the future, more large-scale and high-quality RCTs will be needed as evidence in clinical practice.

## 1 Introduction

Acute coronary syndrome (ACS), the most severe form of cardiovascular disease (Chu et al., 2017) is a major cause of morbidity and mortality worldwide (Eisen et al., 2016; Benjamin et al., 2019). It is characterized by acute myocardial ischemia caused by the disruption of a coronary artery plaque and consequent thrombosis-induced severe coronary artery stenosis or occlusion (Terada et al., 2021). ACS comprises two clinical types: ST-elevation myocardial infarction (STEMI) and non-ST-elevation acute coronary syndrome (NSTE-ACS), with the latter being further divided into unstable angina (UA) and acute non-STEMI (NSTEMI) (Kimura et al., 2019). Percutaneous coronary intervention (PCI) is now a common treatment for ACS (Chacko et al., 2020) that saves myocardial cells by opening diseased vessels and restoring myocardial perfusion. However, PCI may also elicit inflammatory responses and vascular endothelial damage (Tucker et al., 2021). Some PCI-related problems, including no-reflow, ischemia-reperfusion injury, in-stent restenosis, stent thrombosis, and perioperative myocardial injury (PMI), are unavoidable (Cheng et al., 2021). Consequently, the prognosis of PCI in patients with ACS is still not ideal (Cetin et al., 2016). Despite the widespread use of drug-eluting stents, the incidence of major adverse cardiovascular events (MACEs) in the first year after PCI may reach 34.1% (Cai et al., 2019).

Danshen is the dried root and rhizome of *Salvia miltiorrhiza* (Labiaceae) (Xu et al., 2019) and is widely used in Asia as a traditional Chinese medicine to treat various diseases, particularly cardiovascular diseases (Li et al., 2018). Sodium tanshinone IIA sulfonate (STS) is a water-soluble derivative of tanshinone IIA, which is the main lipophilic constituent of Danshen. STS has various pharmacological properties, including anticoagulant, anti-inflammatory, antioxidant, antiviral, anticancer, anti-apoptotic characteristics and iron channel interactions. As such, it can effectively treat a variety of diseases (Zhou et al., 2019). Many studies have found that STS can improve endothelial function and reduce the levels of many inflammatory factors that are associated with the progression of atherosclerosis, such as C-reactive protein (CRP), interleukin-6 (IL-6), tumor necrosis factor-alpha (TNF-α), matrix metalloproteinase-9 (MMP-9), vascular cell adhesion molecule-1 (VCAM-1) and nitric oxide (NO) among others (Li et al., 2017; Ren et al., 2019; Zhu et al., 2022).

In recent years, a large number of clinical trials have shown that STS is beneficial in patients with ACS treated with PCI. However, no firm conclusions could be drawn. Therefore, this systematic review and meta-analysis aimed to methodically evaluate the efficacy and safety of STS on inflammatory factors and vascular endothelial function in patients with ACS undergoing PCI.

## 2 Methods

The study’s review protocol was registered at PROSPERO (No: CRD42022364547, https://www.crd.york.ac.Uk/prospero/) and conducted according to the Cochrane Handbook for Systematic Reviews of Interventions (Cumpston et al., 2022). The study was reported according to the Preferred Reporting Items for Systematic reviews and Meta-Analyses (PRISMA) guidelines (Page et al., 2021).

### 2.1 Data sources and search strategy

A comprehensive search was conducted using the following eight databases: PubMed, the Cochrane Library (CENTRAL), Web of Science, Embase, China National Knowledge Infrastructure (CNKI), Chongqing VIP Information (VIP), and WanFang Data and China Biomedical Literature Database (CBM), from their establishment to 5 October 2022. Two clinical trial registries, ClinicalTrials.gov and Chinese ClinicalTrial Registry (ChiCTR), were also searched. The main search terms were “sodium tanshinone IIA sulfonate” and “acute coronary syndrome.” There were no restrictions on the language, date of publication, or publication status.

### 2.2 Eligibility criteria for included studies

The eligibility criteria of the study conform to the participants, interventions, comparators, outcomes, and study designs (PICOS) principle.

#### 2.2.1 Inclusion criteria

Studies were included based on the following PICOS criteria.

1) Type of participant (P): patients with ACS undergoing PCI therapy. All patients (of any sex, age, or race) met at least one of the current or past definitions or guidelines for ACS established by the World Health Organization, European Society of Cardiology (ESC), American Heart Association/American Heart Association (ACC/AHA), Chinese Society of Cardiology (CSC), and Internal Medicine, or underwent coronary angiography or echocardiography.
2) Types of intervention (I): STS with or without conventional therapy.
3) Types of comparators (C): conventional therapy (CTs), such as antiplatelet agents, statins, renin-angiotensin-aldosterone system blockers, β-blockers, calcium channel blockers, nitrates, and anticoagulant therapy, etc.
4) Types of outcome measures (O): primary outcomes involving inflammatory factors, high-sensitivity C-reactive protein (hs-CRP), tumor necrosis factor-α (TNF-α), matrix metalloproteinase-9 (MMP-9), interleukin-6 (IL-6), superoxide dismutase (SOD), lipid peroxidation (LPO), malondialdehyde (MDA), endothelial function, and nitric oxide (NO); secondary measures relating to major adverse cardiovascular events (MACEs) and adverse events (AEs).
5) Types of studies (S): randomized controlled trials (RCTs) without limits on methods and language.

#### 2.2.2 Exclusion criteria

Studies were excluded if these were 1) non-RCTs, cross-trials, reviews, protocols, case reports, animal experimental studies, conference abstracts and had 2) no full text, incorrect or incomplete data, duplicate publications, extraneous interventions, or relevant results.

### 2.3 Data extraction

Two reviewers independently extracted data according to the afore-mentioned criteria and sorted them into tables. The extracted data included the author’s name, year of publication, sample size, PICOS details, intervention duration, and outcomes. The results were cross-checked during this process, and any differences between the results were resolved after discussion or judged by an arbitrator, if necessary.

### 2.4 Risk of bias assessment

According to the Cochrane Bias Risk Tool 2.0 (RoB2) (Sterne et al., 2019), two reviewers independently assessed the risk of bias in included trials. The following five characteristics will be evaluated: randomization process, deviations from the intended interventions, missing outcome data, measurement of the outcome and selection of the reported result. Each domain was ranked as “low risk of bias,” “some concerns,” or “high risk of bias.” Any disagreement with the assessment was resolved through discussion and consultation with the third author, if necessary. In addition, we used the Graded Grading of Recommendations Assessment, Development, and Evaluation (GRADE) method to assess the quality of evidence.

### 2.5 Data analysis

Review Manager software (RevMan, version 5.4, The Cochrane Collaboration, 2020) was used for data analysis of primary or secondary outcomes, which were then compared between the experimental and control groups. Dichotomous variables were assessed by risk ratios (RR), and continuous variables were analyzed using their mean difference (MD) or standard mean difference (SMD). Between-study heterogeneity among trials was assessed using the Q test and I^2^ statistics. According to the results of the heterogeneity test, the random effects model or fixed effects model was selected for data analysis. When no statistical heterogeneity was identified (heterogeneity test, *p* ≥ 0.10, or I^2^ ≤ 50%), the fixed-effects model was selected; otherwise, the random-effects model was used. Outcomes were calculated using *p* values, and *p* < 0.05 was considered statistically significant. In addition, meta-regression analysis was performed to explore the potential sources of heterogeneity. Statistically significant factors obtained through meta-regression analysis will be used as grouping indicators for subgroup analysis. Sensitivity analysis was performed by omitting each study at a time to assess the stability of the combined results. Funnel plots, Begger’s test, and Egger’s test were used to detect publication bias.

### 2.6 Certainty assessment

Two reviewers independently assessed the certainty of the evidence using the GRADE method (Balshem et al., 2011) and rated the certainty of the evidence as “high,” “medium,” “low” or “very low.” Certainty may be downgraded by five GRADE factors (study limitations, effect consistency, imprecision, indirection, and publication bias) and upgraded for three reasons (large effect magnitude, influence of dose-response gradient, and reasonable residual confounding).

## 3 Results

### 3.1 Study selection

**FIGURE 1:**
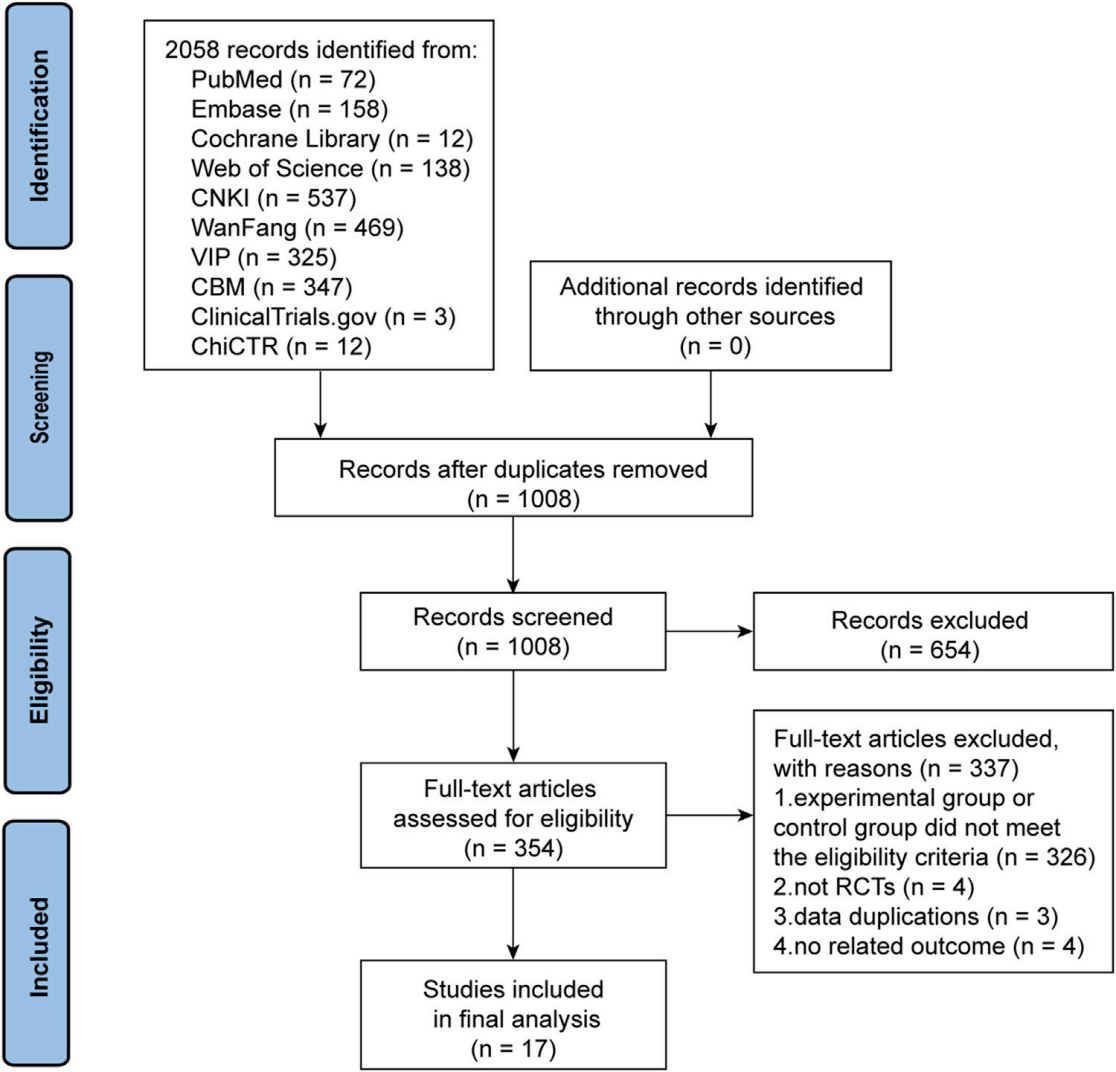
The PRISMA flow chart of the literature screening and selection process.

The initial search yielded 2,073 articles. After deleting duplicate literature, 1,008 articles were screened for title and abstract. A total of 654 articles were subsequently excluded and 354 articles were then screened in detail. After reading the full text of the remaining 354 articles, 326 trials in the experimental group or control group failed to meet the eligibility criteria. Four trials did not belong to RCTs (Ye, 2019; Li, 2020; Wen and LYU, 2020; Zhao et al., 2022), three trials had duplicate data (Luan, 2012; Pang, 2016; He and Wang, 2019), and another four trials did not include outcomes that met the criteria (Lin, 2012; Mao et al., 2015a; Mao et al., 2015b; Zhou et al., 2016). Ultimately, 17 trials (Du and Wang, 2011; Luan and Pan, 2012; Wu et al., 2015; He, 2016; Li, 2016; Chen et al., 2017; Lin et al., 2017; Du et al., 2018; Luo, 2018; Pang et al., 2019; Tao et al., 2019; Zhang and Jin, 2019; Lian, 2020; Lu et al., 2021; Mao et al., 2021; Zheng et al., 2021; Yu et al., 2022) were deemed qualified for the meta-analysis. The PRISMA flow chart of the trial selection process is shown in Figure 1.

### 3.2 Study characteristics

The 17 RCTs included were conducted in various hospitals in China, with sample sizes ranging from 58 to 100, including two multicenter studies (Lian, 2020; Mao et al., 2021). A total of 1,802 hospitalized patients were included (908 in the experimental group and 894 in the control group). With the exception of five trials that did not report age and sex composition, most of the studies recruited more male patients. All research interventions included STS in combination with CTs. The STS doses range from 20 to 40 mg/day intravenously, with treatment durations ranging from 5 to 40 days. Only three trials (Wu et al., 2015; Zheng et al., 2021; Yu et al., 2022) did not report the dose or duration of treatment.

Most studies reported the results of inflammatory factors and vascular endothelial function in detail, among which the more commonly used indicators were analyzed. Ten trials (Du and Wang, 2011; He, 2016; Li, 2016; Du et al., 2018; Luo, 2018; Zhang and Jin, 2019; Lian, 2020; Lu et al., 2021; Mao et al., 2021; Yu et al., 2022) reported MACE information. In addition, ten trials reported adverse events, of which four (Chen et al., 2017; Lin et al., 2017; Luo, 2018; Tao et al., 2019) reported no adverse events and the remaining six (He, 2016; Li, 2016; Pang et al., 2019; Lian, 2020; Zheng et al., 2021; Yu et al., 2022) reported a total of 28 adverse events. The basic characteristics of the included RCTs are shown in Table 1.

**TABLE 1:** Characteristics of the included RCTs and the detail of PICOS.

Study ID	Sample size (E/C)	Age (E/C)	Male (%) (E/C)	Interventions		Duration	Outcome
				Experiment group	Control group		
Du and Wang (2011)	26/22	unclear	unclear	STS 80 mg qd + CTs	CTs	14 days	⑨
Luan and Pan (2012)	20/20	unclear	unclear	STS 50 mg qd + CTs	CTs	7 days	②④⑤⑦⑧
Wu et al. (2015)	40/40	66.44 ± 7.78/66.71 ± 8.31	22(55.00%)/25(62.50%)	STS 60 mg bid + CTs	CTs	unclear	①⑤
He (2016)	30/30	59.97 ± 11.28/61.63 ± 12.01	24(80.00%)/23(76.67%)	STS 80 mg qd + CTs	CTs	5 days	①⑨⑩
Li (2016)	35/37	66.89 ± 10.08/66.92 ± 9.25	22(62.86%)/29(78.38%)	STS 80 mg qd + CTs	CTs	5 days	①⑨⑩
Chen et al. (2017)	30/29	unclear	unclear	STS 80 mg qd + CTs	CTs	7 days	⑩
Lin et al. (2017)	49/49	55.98 ± 5.49/56.44 ± 5.86	30(61.22%)/32(65.31%)	STS 50 mg qd + CTS	CTs	14 days	①②③⑤⑥⑦⑩
Du et al. (2018)	75/75	54.00 ± 8.00/53.00 ± 9.20	54(72.00%)/52(69.33%)	STS 80 mg qd + CTs	CTs	14 days	⑨
Luo (2018)	43/43	57.85 ± 4.61/58.01 ± 5.02	24(55.81%)/23(53.49%)	STS 80 mg qd + CTs	CTs	7 days	⑨⑩
Pang et al. (2019)	31/31	65.77 ± 10.30/63.06 ± 8.78	21(67.74%)/22(70.97%)	STS 80 mg qd + CTs	CTs	5 days	①⑤⑧⑩
Tao et al. (2019)	40/40	58.70 ± 7.80/58.00 ± 6.90	22(55.00%)/26(65.00%)	STS 80 mg qd + CTs	CTs	40 days	②③⑤⑥⑦⑩
Zhang and Jin (2019)	102/102	56.00 ± 5.30/54.00 ± 6.30	52(50.98%)/50(49.02%)	STS 50 mg qd + CTs	CTs	7 days	②④⑤⑦⑧⑨
Lian (2020)	34/35	60.80 ± 8.30/64.60 ± 8.70	26(76.47%)/27(77.14%)	STS 80 mg qd + CTs	CTs	5 days	①⑨⑩
Lu et al. (2021)	100/100	58.06 ± 4.84/58.13 ± 5.16	58(58.00%)/57(57.00%)	STS 80 mg qd + Alprostadil 10 mg qd + CTs	Alprostadil 10 mg qd + CTs	7 days	⑨
Mao et al. (2021)	192/180	62.49 ± 10.20/64.15 ± 10.00	134(69.79%)/129(71.67%)	STS 80 mg qd + CTs	CTs	5 days	⑨
Zheng et al. (2021)	20/20	57.33 ± 3.91/58.21 ± 3.85	12(60.00%)/11(55.00%)	STS 80 mg qd + CTs	CTs	unclear	①⑩
Yu et al. (2022)	41/41	59.26 ± 8.19/57.81 ± 8.06	24(58.54%)/27(65.85%)	STS 40–80 mg qd + Carvedilol 12.5 mg bid + CTs	Carvedilol 12.5 mg bid + CTs	32 days	⑨⑩

E/C, Experimental group/Control group; STS, Sodium Tanshinone IIA, sulfonate injection; CTs, conventional therapy; ①hs-CRP: high-sensitivity C-reactive protein; ②TNF-α: tumor necrosis factor-α; ③MMP-9: matrix metalloproteinase-9; ④IL-6: interleukin-6; ⑤SOD: superoxide dismutase; ⑥LPO: lipid peroxidation; ⑦MDA: malondialdehyde; ⑧NO: nitric oxide; ⑨MACEs: major adverse cardiovascular events; ⑩AEs: adverse events.

### 3.3 Risk of bias

**FIGURE 2:**
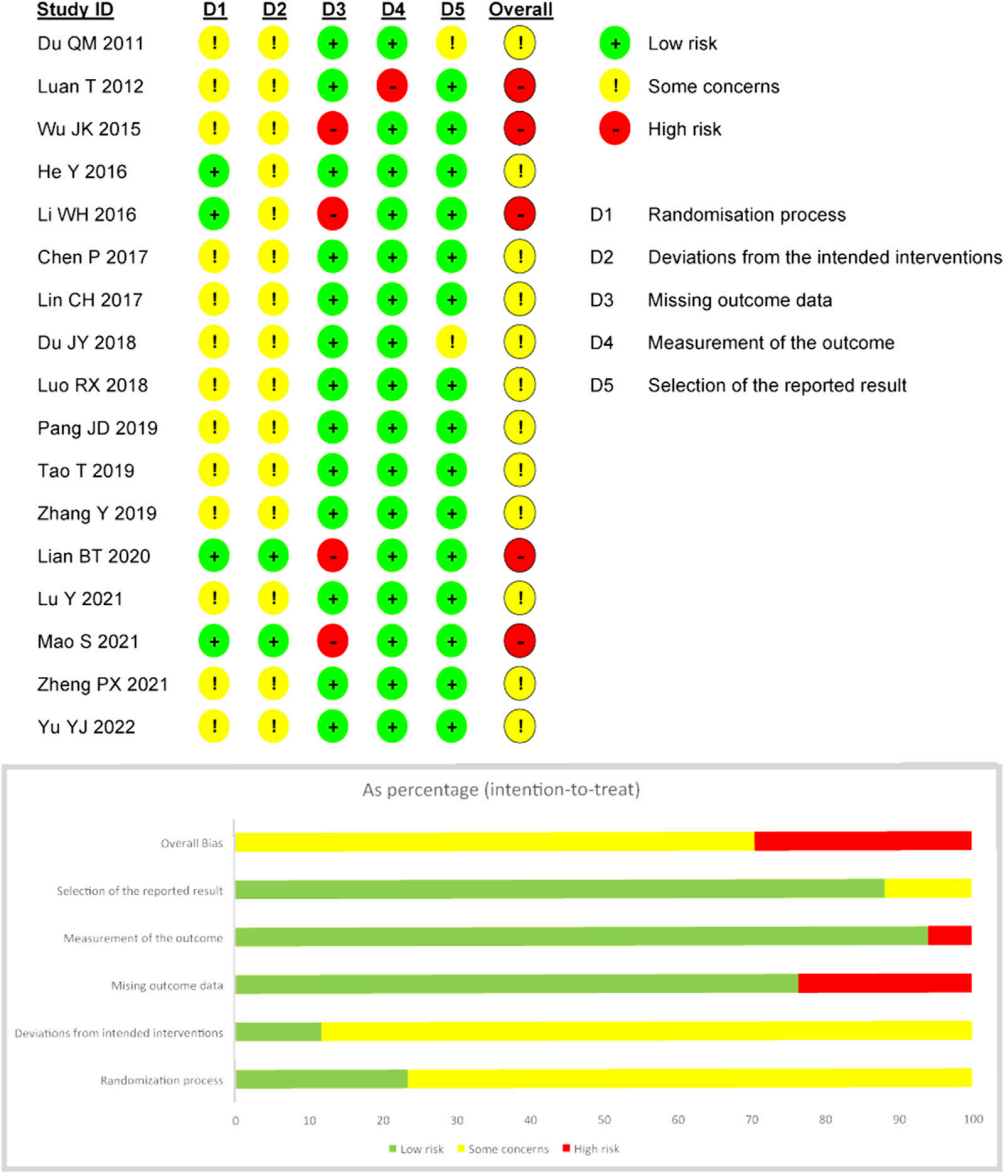
Risk of bias summary.

All of the trials mentioned randomization, 12 trials (Wu et al., 2015; He, 2016; Li, 2016; Chen et al., 2017; Lin et al., 2017; Luo, 2018; Tao et al., 2019; Zhang and Jin, 2019; Lian, 2020; Lu et al., 2021; Mao et al., 2021; Yu et al., 2022) mentioned the use of random sequence methods such as random number tables, computer-generated random numbers, or central random systems, and four of them (He, 2016; Li, 2016; Lian, 2020; Mao et al., 2021) also mentioned methods of assigning concealment and were therefore considered “low risk.” For “bias due to deviating from expected interventions,” two trials (Lian, 2020; Mao et al., 2021) were rated “low risk” because they used blinding for subjects, operators, and outcome evaluators. Four trials (Wu et al., 2015; Li, 2016; Lian, 2020; Mao et al., 2021) did not explicitly explain the absence of follow-up, so “bias due to missing outcome data” was rated as “high risk.” One trial (Wu et al., 2015) was rated “high risk” for “bias due to measurement of the outcome” because it did not blind the evaluator, and the remaining studies should be considered “low risk” because of the objectivity of the outcome measures. In addition, the “bias in the selection of the reported result” in two trials (Du and Wang, 2011; Du et al., 2018) was rated as “some concerns” because planned outcomes were not explicitly mentioned in the prespecified protocol, while the remaining trials were assessed as “low risk” because their outcome measurement and analysis were consistent with the prespecified protocol. Based on the assessment of the above five areas, the overall bias of five trials (Luan and Pan, 2012; Wu et al., 2015; Li, 2016; Lian, 2020; Mao et al., 2021) was rated as “high risk of bias,” while the overall bias of other trials was rated as “some concerns.” The specific information of bias risk assessment is shown in Figure 2.

### 3.4 Overall results of meta-analysis

Except for MACEs, AEs, and two indicators (MMP-9 and LPO), the meta-analyses showed a high degree of heterogeneity among the studies. Therefore, the fixed-effect model was used for the meta-analysis of MACEs, AEs, MMP-9, and LPO, while the random-effects model was used for the meta-analyses of other indicators.

#### 3.4.1 hs-CRP

**FIGURE 3:**
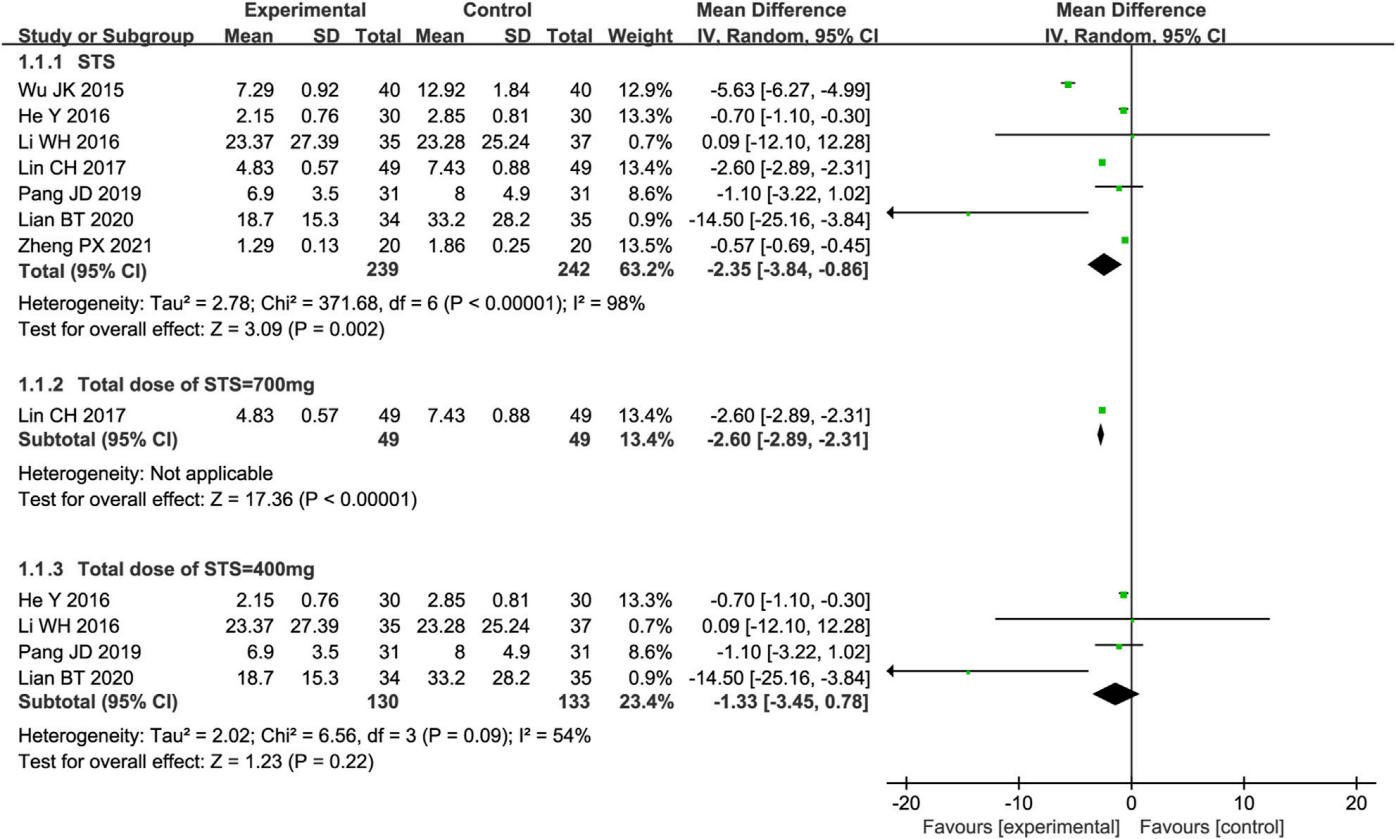
Forest plot of hs-CRP.

A meta-analysis of seven trials showed that STS significantly reduced hs-CRP levels in patients [MD = −2.35, 95% CI (−3.84, −0.86), *p* = 0.002]. Due to significant clinical heterogeneity (*p* < 0.00001, I^2^ = 98%), meta-regression analysis was performed to identify possible sources of high heterogeneity. Meta-regression analysis determined that heterogeneity was independent of the intervention time point and test method but was related to the total dose of STS (*p* < 0.05, Supplementary Figure S1). Therefore, a subgroup analysis was performed based on the total dose of STS. The results showed that both a total dose of 700 mg [MD = −2.60, 95%CI (−2.89, −2.31), *p* < 0.00001] and a total dose of 400 mg [MD = −1.33, 95%CI (−3.45, 0.78), *p* = 0.22] of STS significantly reduced hs-CRP levels compared with the control group. This reduced considerably heterogeneity between studies (*p* = 0.09, I^2^ = 54%). Because the total STS dose was not reported in the two studies, a subgroup analysis for these studies was not possible. The results are shown in Figure 3.

#### 3.4.2 TNF-α

**FIGURE 4:**
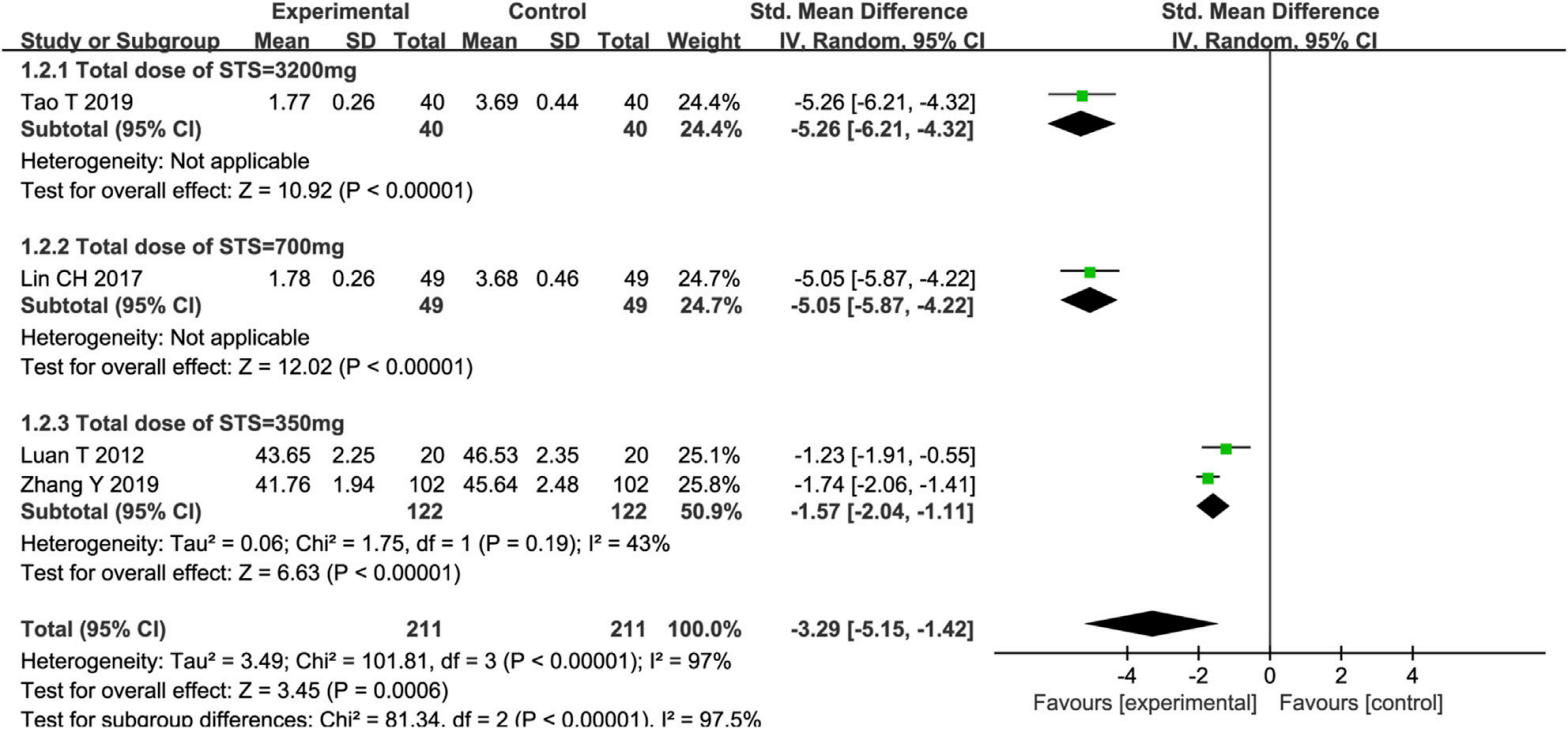
Forest plot of TNF-α.

Four trials reported on TNF-α, and the meta-analysis results showed that STS significantly reduced TNF-α levels in patients [SMD = −3.29, 95%CI (−5.15, −1.42), *p* = 0,006]. Among the four trials, the intervention time point of STS and the detection methods of TNF-α were consistent. Meta-regression analysis determined that significant clinical heterogeneity (*p* < 0.00001, I^2^ = 97%) was related to the total dose of STS (*p* < 0.05, Supplementary Figure S2). A subgroup analysis based on the total dose of STS showed that, compared with the control group, different total doses of STS also significantly reduced TNF-α levels (Total dose of STS = 3200 mg: SMD = −5.26, 95%CI (−6.21, −4.32), *p* < 0.00001; Total dose of STS = 700 mg: SMD = −5.05, 95%CI (−5.87, −4.22), *p* < 0.00001; Total dose of STS = 400 mg: SMD = −1.57, 95%CI (−2.04, −1.11), *p* < 0.00001] compared with the control group. The heterogeneity between studies was significantly reduced (*p* = 0.19, I^2^ = 43%). The results are presented in Figure 4.

#### 3.4.3 MMP-9

**FIGURE 5:**
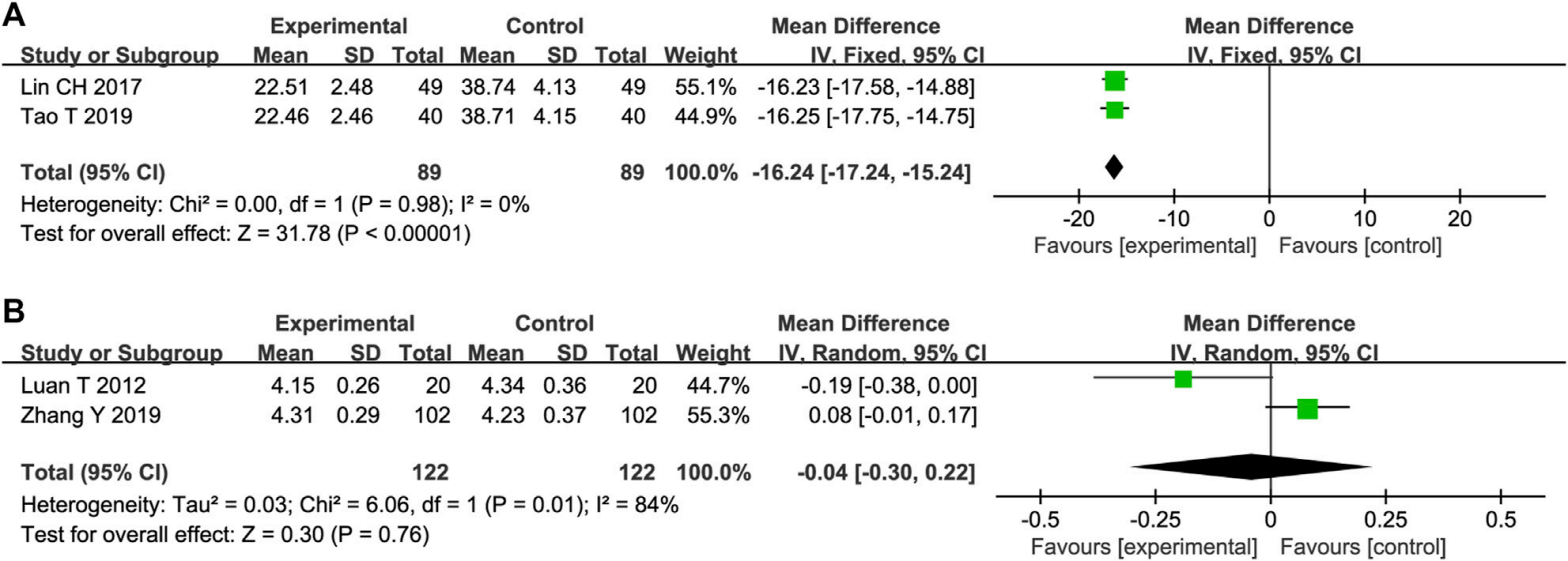
Forest plot of MMP-9 **(A)** and IL-6 **(B)**.

Two trials reported the effects of STS on MMP-9 expression. The heterogeneity between these studies was insignificant (*p* = 0.98, I^2^ = 0%); therefore, a fixed-effects model was adopted. The results showed that STS was superior to the control group in reducing MMP-9 levels [MD = −16.24, 95%CI (−17.24, −15.24), *p* < 0.00001], Figure 5A).

#### 3.4.4 IL-6

Two trials reported the effects of STS on IL-6 levels. The heterogeneity of the two studies was significant (*p* = 0.01, I^2^ = 84%). In addition, the pooled results of the two trials showed that there was no statistical difference in reducing IL-6 between the two groups [MD = −0.04, 95%CI (−0.30, 0.22), *p* = 0.76, Figure 5B].

#### 3.4.5 SOD

**FIGURE 6:**
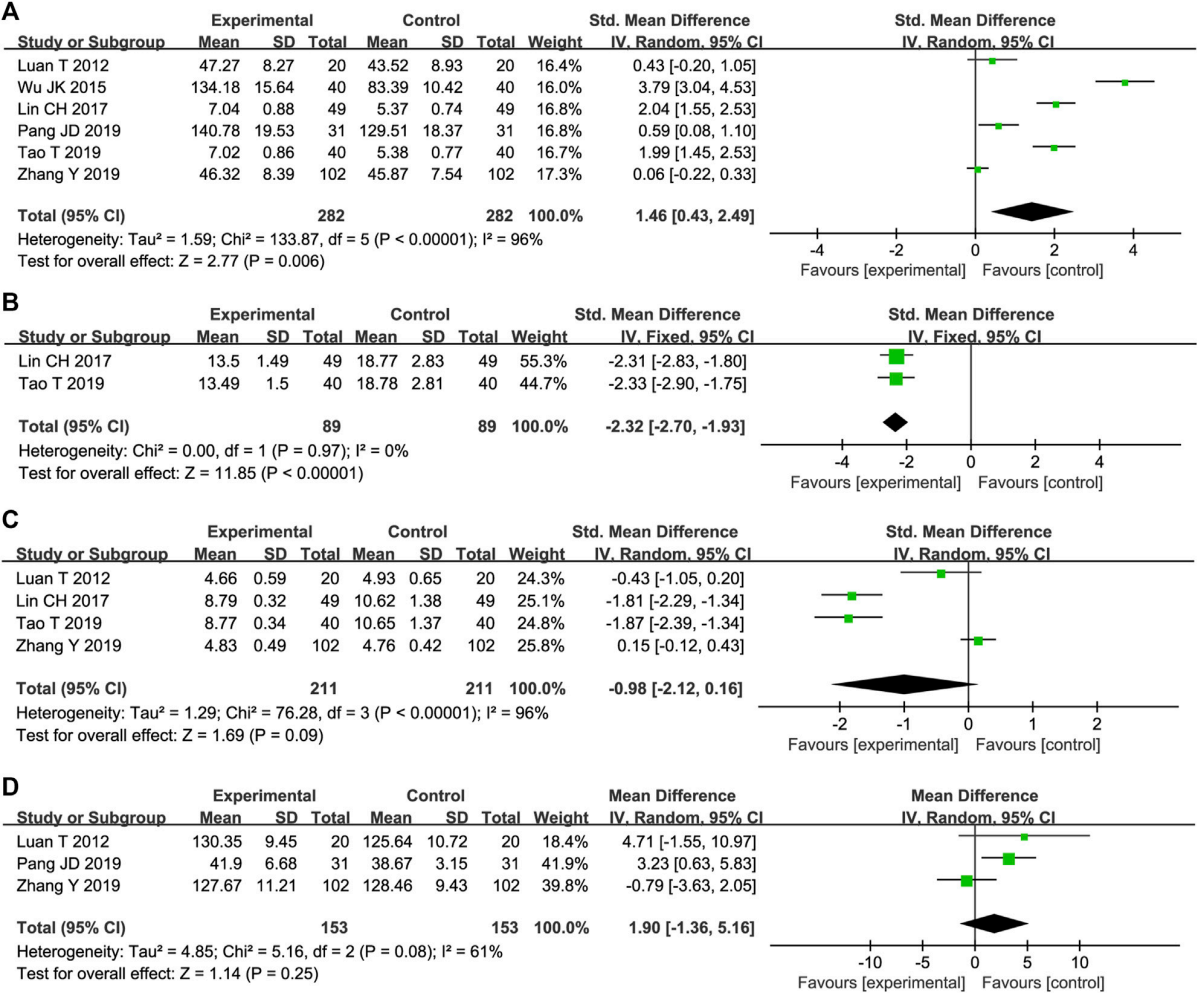
Forest plot of SOD **(A)**, LPO **(B)**, MDA **(C)** and NO **(D)**.

Six trials reported STS effects on SOD levels. The results of the meta-analysis showed that STS versus conventional drug therapy significantly increased SOD levels [SMD = 1.46, 95%CI (0.43, 2.49), *p* = 0,006], Figure 6A). Due to the high heterogeneity among studies (*p* < 0.00001, I^2^ = 96%), sensitivity analysis was conducted by excluding studies individually. After removing one reported trial (Wu et al., 2015), the MD was used to merge the results because the units of the remaining research results were all similar. As shown in Supplementary Figure S3, the heterogeneity was significantly reduced to 31%, and a fixed-effect model was adopted. As shown in Table 1, compared with other studies, this trial did not mention the course of treatment, and the STS dosage used was the highest dose possible, which may have resulted in high heterogeneity.

#### 3.4.6 LPO

STS effects on LPO were reported in two trials. The heterogeneity between them was insignificant (*p* = 0.97, I^2^ = 0%); therefore, a fixed-effects model was adopted. The results showed that STS was superior to the control group in reducing LPO [MD = −2.32, 95%CI (−2.70, −1.93), *p* < 0.00001], Figure 6B).

#### 3.4.7 MDA

As shown in Figure 6C, MDA levels were reported in four trials. The results of two trials showed that STS was superior to CTs in reducing MDA [SMD = −1.81, 95%CI (−2.29, −1.34), *p* < 0.00001]; SMD = −1.87, 95%CI (−2.39, −1.34), *p* < 0.00001]. However, the pooled results of the four trials showed that there was no statistical difference in MDA reduction between the two groups [SMD = −0.98, 95%CI (−2.12, 0.16), *p* < 0.00001].

#### 3.4.8 NO

STS effects on NO were reported in three trials. The results of one trial showed that STS was superior to the control group in increasing NO levels [MD = 3.23, 95%CI (0.63, 5.83), *p* = 0.01]. However, the pooled results of the three trials showed that there was no statistical difference in reducing NO between the two groups [MD = 1.90, 95%CI (−1.36, 5.16), *p* = 0.25, Figure 6D].

#### 3.4.9 MACEs

**FIGURE 7:**
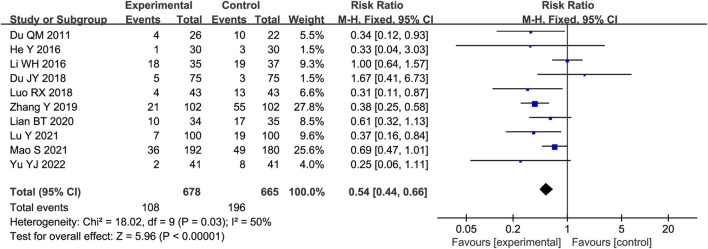
Forest plot of major adverse cardiovascular events (MACEs).

Ten trials reported the occurrence of MACEs in STS-treated patients. As heterogeneity among the studies was insignificant (*p* = 0.03, I^2^ = 50%), a fixed-effects model was used. As shown in Figure 7, the results of the meta-analysis indicated that STS reduced the occurrence of MACE compared with the control group [RR = 0.54, 95%CI (0.44, 0.66), *p* < 0.00001].

#### 3.4.10 AEs

**FIGURE 8:**
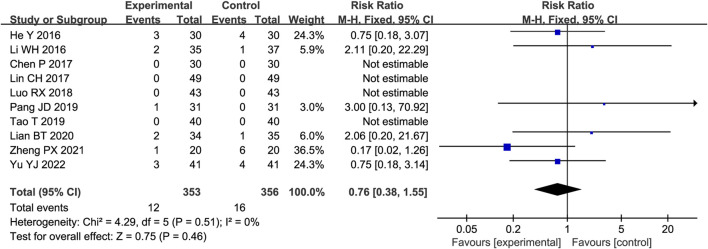
Forest plot of major adverse events (AEs).

Ten trials reported AEs, four of which reported no adverse reactions in either group, while the remaining studies reported adverse reactions in both groups. As shown in Figure 8, there was little heterogeneity among the studies (*p* = 0.51, I^2^ = 0%); therefore, the fixed-effect model was adopted. Meta-analysis showed no significant difference in adverse events between the two groups (RR = 0.76, 95%CI [0.38, 1.55], *p* = 0.46).

### 3.5 Publication bias

**FIGURE 9:**
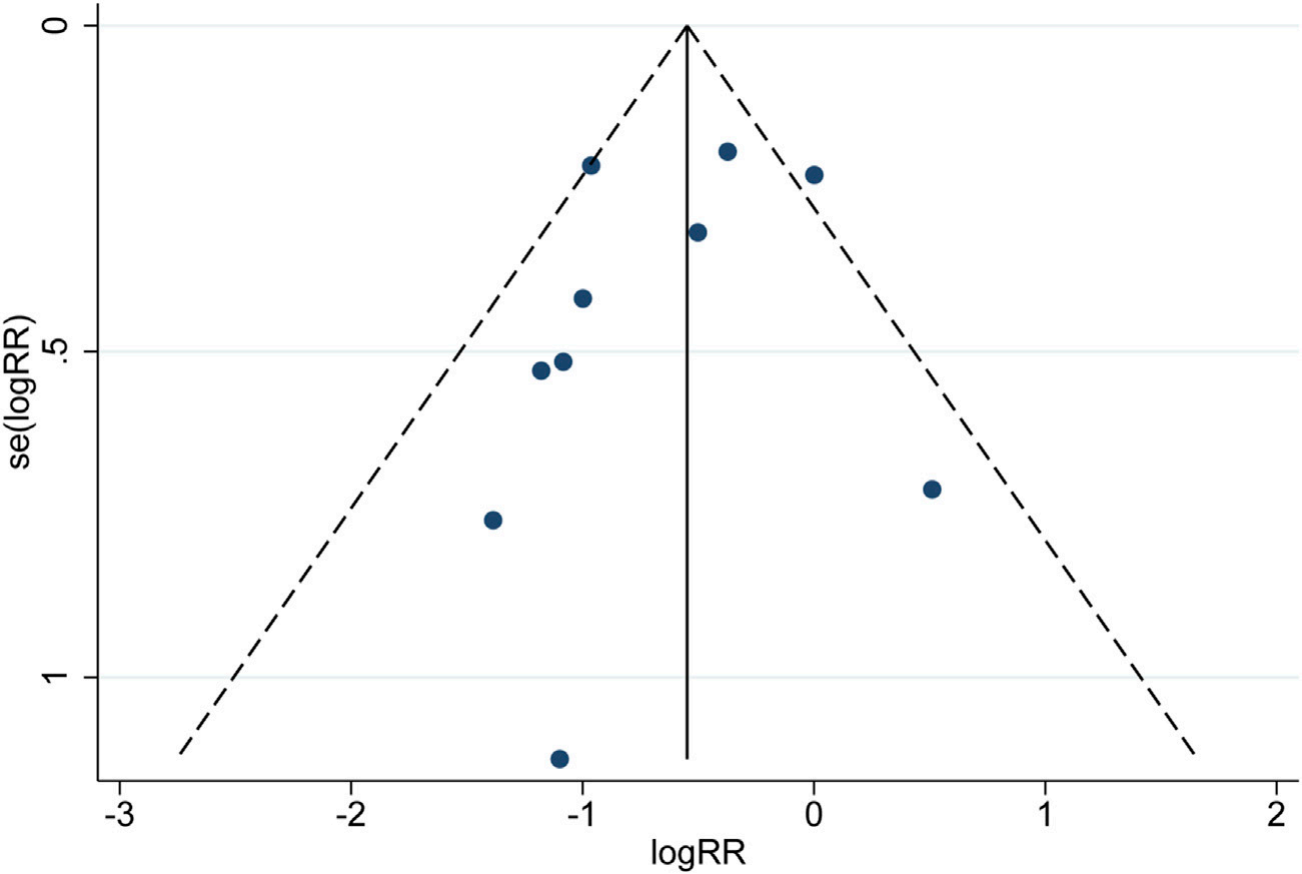
Funnel plot of incidence of major adverse cardiovascular events (MACEs).

Publication bias for MACEs was evaluated using a funnel plot (Figure 9). Begg’s and Egger’s tests showed that the *p* values were all greater than 0.05 (Begg, z = −0.450, *p* = 0.721; Egger, z = −0.890, *p* = 0.397), suggesting that the publication bias associated with MACEs was not significant. Although the probability of publication bias is statistically tiny, we still believe that the possibility of publication bias is more significant because the included trials are all in Chinese except for two in English. Moreover, positive results are more likely to be published.

### 3.6 GRADE assessment

The certainty of evidence on outcomes of the meta-analysis was assessed using the GRADE system, which showed that the evidentiary quality varied from “very low” to “moderate.” The main reasons for downgrading were a high risk of bias, inconsistency, and imprecision. The GRADE profiles are shown in Supplementary Table S1, S2.

## 4 Discussion

In this systematic review, 17 RCTs involving 1,802 subjects were analyzed in order to assess the effects of STS on inflammatory factors and vascular endothelial function in patients with ACS treated with PCI. The results showed that STS could significantly reduce the levels of hs-CRP, TNF-α, MMP-9, and LPO and increase the level of SOD. However, there is insufficient evidence that STS inhibits IL-6, MDA, and NO expression. Regarding the occurrence of MACEs, the STS group had a reduced occurrence of adverse cardiac events. There were no statistically significant differences in the AEs between the STS and control groups. STS therapy may be safe and effective in reducing inflammatory factors and improving endothelial function in patients with ACS treated with PCI and has great potential as an adjunct drug to improve patient prognosis. However, owing to the low overall quality of the current RCTs, the above conclusions must be verified through high-quality studies with larger sample sizes.

Although PCI is quite effective at restoring coronary blood flow, it is an invasive procedure that is very likely to cause vascular endothelial injury (Veronesi et al., 2017) and may be associated with a number of pathological mechanisms (Kang et al., 2022), including platelet aggregation and inflammation. This can lead to bleeding, stent restenosis, major adverse cardiac events, and poor prognosis, making PCI challenging for patients with ACS. Traditional Chinese medicine has some limitations due to a lack of adequate basic research, but the increasingly effective evidence-based practice has made it an effective treatment for many diseases (Zou et al., 2018). As a commonly used injectable agent for cardiovascular diseases, STS has been frequently studied to explore the mechanisms related to improving inflammatory factors and vascular endothelial function. Inflammatory factors such as hs-CRP, TNF-α, IL-6, and MMP-9 are actively involved in vascular inflammatory responses and the development of coronary atherosclerosis (Gao et al., 2018). Increasing evidence suggests that inflammatory factors play an essential role in the occurrence and development of heart disease (Ma et al., 2017). Inflammation interacts with oxidative stress (Steven et al., 2019) and increases the production of reactive oxygen species (ROS), which in turn, increases inflammation in a vicious cycle. SOD is an important antioxidant enzyme, and LPO and MDA are peroxide products (Yu and Zhou, 2022). Reducing MDA and LPO levels and increasing SOD levels can reduce oxidative stress (Hong et al., 2020). Although this study lacks strong evidence on the results of IL-6 and MDA, some studies have shown that STS can effectively reduce the expression of TNF-α, IL-6, MMP-9, chloride intracellular channel 1 (CLIC1), vascular cell adhesion molecule 1 (VCAM-1), and other inflammatory factors in atherosclerotic mice, whereas STS can reduce the production of MDA and increase the activity of SOD (Ji et al., 2017; Meng et al., 2018; Liu et al., 2020). This activity may mediate STS antioxidant and anti-inflammatory properties by inhibiting CLIC1 expression and membrane translocation (Zhu et al., 2017). Some studies have also shown that this process is achieved by blocking the activation of the mitogen-activated kinase (MAPK)/hypoxia-inducible factor-1(HIF-1α) signaling pathway (Guan et al., 2018). NO also plays a crucial role in atherosclerosis by controlling the cellular processes of vascular smooth muscle cells to maintain endothelial balance (Umman et al., 2015). STS exerts its effects on these vascular endothelial cells through various functions. Although this study lacks strong evidence for the effect of STS on NO reduction, some studies have proposed that STS promotes NO production and inhibits heat stress-induced apoptosis of human umbilical vein endothelial cells (HUVECs) through the PI3K/AKT/eNOS pathway (Cheng et al., 2017).

This is the first systematic review to report that STS improves inflammatory factors and vascular endothelial function in patients with ACS treated with PCI by strictly following the PRISMA guidelines, and applying the GRADE criteria to determine certainty in estimates of significant outcome effects. However, this study has certain limitations. First, due to the small number of included studies and their low to moderate quality, rigorously designed trials declared under CONSORT protocols are required to verify the effectiveness of STS as adjunctive therapy for ACS patients undergoing PCI. Second, most trials did not specify methodological details such as random patterns, allocation hiding, and blindness, which greatly weakened the credibility of the evidence. Finally, the treatment duration and STS dosages varied greatly. Owing to the small number of studies that were included, we only conducted a subgroup analysis for two inflammatory factors, hs-CRP and TNF-α. Finally, the heterogeneity and publication bias of the results were considerably significant, thus requiring a cautious interpretation of the sources and results.

This systematic review provides a small amount of evidence that STS improves the prognosis of ACS patients treated with PCI. STS has the potential to be a promising adjunctive therapy for improving PCI treatment in patients with ACS that clinicians can consider. Nevertheless, further attention should be given to the improvement of STS safety evaluations. The long-term efficacy of STS should also be explored in future studies.

## 5 Conclusion

STS can safely and effectively reduce the levels of hs-CRP, TNF-α, MMP-9, and LPO and increase the levels of SOD in patients with ACS treated with PCI. It can also reduce the incidence of adverse cardiovascular events. However, these findings require careful consideration due to the small number of studies, high risk of bias, and low to moderate evidence. In the future, more large-scale and high-quality RCTs will be needed as evidence in clinical practice.

## Data Availability

The original contributions presented in the study are included in the article/Supplementary Material, further inquiries can be directed to the corresponding author.
