# Supplementary material for: Effects of sodium tanshinone IIA sulfonate injection on inflammatory factors and vascular endothelial function in patients with acute coronary syndrome undergoing percutaneous coronary intervention: A systematic review and meta-analysis of randomized clinical trials
Source: Front Pharmacol. 2023 Mar 7;14:1144419. doi: 10.3389/fphar.2023.1144419 (PMC10027702; doi:10.3389/fphar.2023.1144419)
Supplement: Supplementary file 1 [file Table1.DOCX]

Supplementary Material

## Supplementary Figures


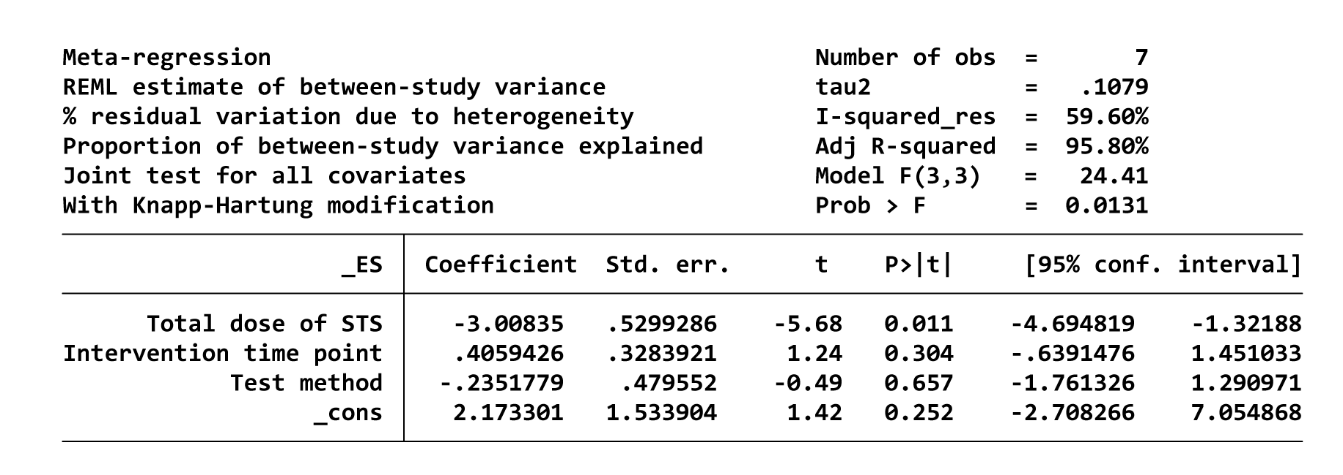


**Supplementary Figure S1.** Meta-regression analysis of hs-CRP


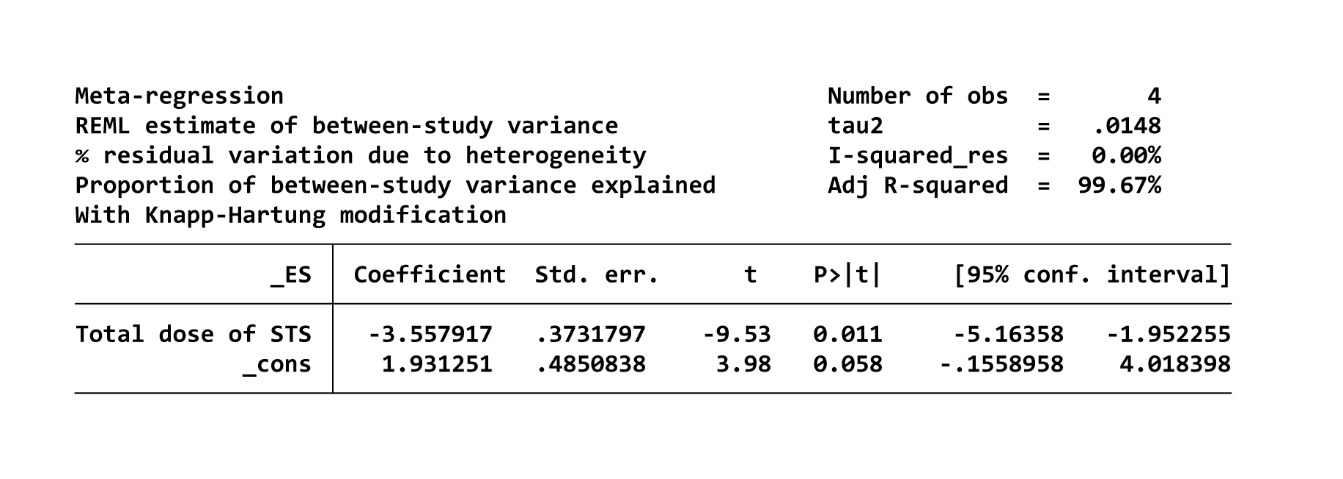


**Supplementary Figure S2.** Meta-regression analysis of TNF-α


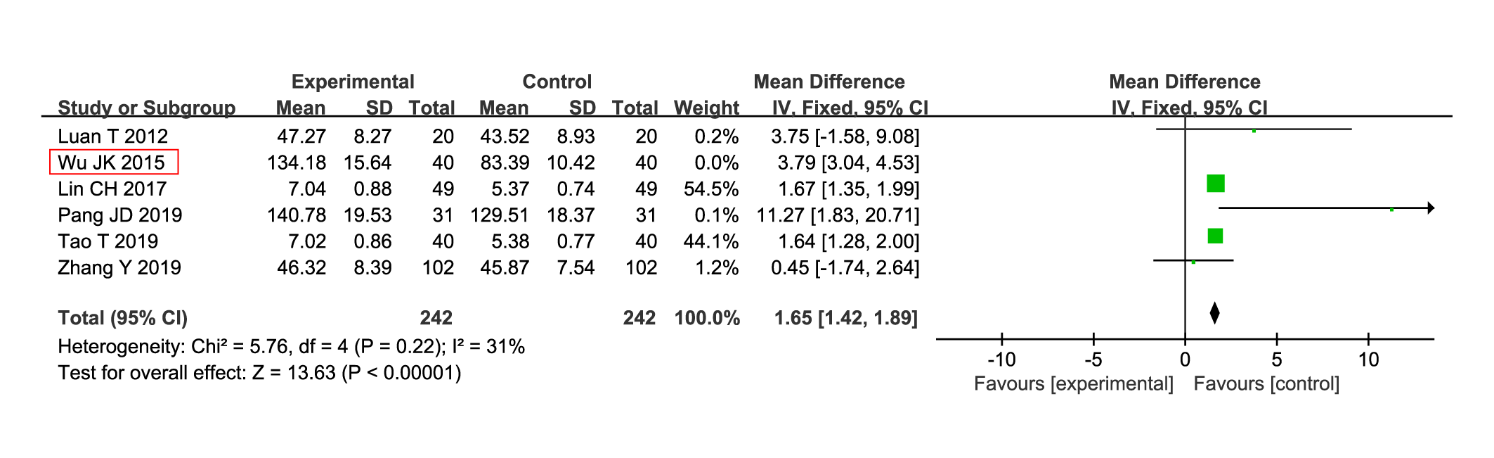


**Supplementary Figure S3.** Sensitivity analysis of SOD
