# Supplementary material for: Effects of sodium tanshinone IIA sulfonate injection on inflammatory factors and vascular endothelial function in patients with acute coronary syndrome undergoing percutaneous coronary intervention: A systematic review and meta-analysis of randomized clinical trials
Source: Front Pharmacol. 2023 Mar 7;14:1144419. doi: 10.3389/fphar.2023.1144419 (PMC10027702; doi:10.3389/fphar.2023.1144419)
Supplement: Supplementary file 2 [file Table2.DOCX]

Supplementary Material

## Supplementary Tables

| **Quality assessment** | | | | | | | **No of patients** | | **Effect** | **Quality** | **Importance** |
| --- | --- | --- | --- | --- | --- | --- | --- | --- | --- | --- | --- |
|  |  |  |  |  |  |  |  |  |  |  |  |
| **(outcomes)No of studies** | **Design** | **Risk of bias** | **Inconsistency** | **Indirectness** | **Imprecision** | **Other considerations** | **STS** | **non-STS** | **Absolute** |  |  |
| (hs-CRP) 7 | randomised trials | no serious risk of bias | very serious | no serious indirectness | no serious imprecision | none | 239 | 242 | MD 2.35 lower (3.48 to 0.86 lower) | ⊕⊕OO LOW | IMPORTANT |
| (TNF-α) 4 | randomised trials | serious | very serious | no serious indirectness | no serious imprecision | none | 211 | 211 | SMD 3.29 lower (5.15 to 1.42 lower) | ⊕OOO VERY LOW | IMPORTANT |
| (MMP-9) 2 | randomised trials | serious | no serious inconsistency | no serious indirectness | serious | none | 89 | 89 | MD 16.24 lower (17.24 to 15.24 lower) | ⊕⊕OO LOW | IMPORTANT |
| (IL-6) 2 | randomised trials | serious | very serious | no serious indirectness | very serious | none | 122 | 122 | MD 0.04 lower (0.3 lower to 0.22 higher) | ⊕OOO VERY LOW | IMPORTANT |
| (SOD) 6 | randomised trials | serious | very serious | no serious indirectness | no serious imprecision | none | 282 | 282 | MD 1.73 higher (1.5 to 1.97 higher) | ⊕OOO VERY LOW | IMPORTANT |
| (LPO) 2 | randomised trials | serious | no serious inconsistency | no serious indirectness | serious | none | 89 | 89 | SMD 2.32 lower (2.7 to 1.93 lower) | ⊕⊕OO LOW | IMPORTANT |
| (MDA) 4 | randomised trials | serious | very serious | no serious indirectness | serious | none | 211 | 211 | SMD 0.98 lower (2.12 lower to 0.16 higher) | ⊕OOO VERY LOW | IMPORTANT |
| (NO) 3 | randomised trials | serious | serious | no serious indirectness | very serious | none | 153 | 153 | MD 1.9 higher (1.36 lower to 5.16 higher) | ⊕OOO VERY LOW | IMPORTANT |

**Supplementary Table S1.** The summary findings by the grading recommendations assessment, development, and evaluation (GRADE) methods.

| **Quality assessment** | | | | | | | **No of patients** | | **Effect** | | **Quality** | **Importance** |
| --- | --- | --- | --- | --- | --- | --- | --- | --- | --- | --- | --- | --- |
|  |  |  |  |  |  |  |  |  |  |  |  |  |
| **(outcomes)**  **No of studies** | **Design** | **Risk of bias** | **Inconsistency** | **Indirectness** | **Imprecision** | **Other considerations** | **STS** | **non-STS** | **Relative (95% CI)** | **Absolute** |  |  |
| (MACEs) 10 | randomised trials | no serious risk of bias | no serious inconsistency | serious | no serious imprecision | none | 108/678 (15.9%) | 196/665 (29.5%) | RR 0.54 (0.44 to 0.66) | 136 fewer per 1000 (from 100 fewer to 165 fewer) | ⊕⊕⊕O MODERATE | IMPORTANT |
| (AEs) 10 | randomised trials | no serious risk of bias | no serious inconsistency | serious | serious | none | 12/353 (3.4%) | 16/356 (4.5%) | RR 0.76 (0.38 to 1.55) | 11 fewer per 1000 (from 28 fewer to 25 more) | ⊕⊕OO LOW | IMPORTANT |

**Supplementary Table S2.** The summary findings by the grading recommendations assessment, development, and evaluation (GRADE) methods.
